# Arsenic, Cadmium, Uranium and Vanadium Produce Shared and Distinct Gene Expression Signatures in Primary Human Renal Cells

**DOI:** 10.3390/toxics14080648

**Published:** 2026-07-23

**Authors:** Jodi Schilz, Erica Dashner-Titus, Karen Torczynski, Laurie Hudson

**Affiliations:** 1 Division of Physical Therapy, School of Medicine, University of New Mexico, Albuquerque, NM 87131, USA; jschilz@salud.unm.edu; 2 Department of Pharmaceutical Sciences, College of Pharmacy, University of New Mexico, Albuquerque, NM 87131, USA; edashner@salud.unm.edu (E.D.-T.); ktorczynski@salud.unm.edu (K.T.)

**Keywords:** metal toxicity, renal epithelial cells, transcriptomics, arsenic, cadmium, vanadium, uranium

## Abstract

Many metals in the environment are nephrotoxic. To better understand both similar and dissimilar responses to different metals, we compared RNA sequencing data from primary human proximal tubule epithelial kidney cells treated with arsenic, cadmium, vanadium, or uranium for 6 or 24 h. At 24 h post-treatment, arsenic- or cadmium-treated cells shared processes related to metal stress response and detoxification. Distinct characteristics of arsenic exposure included terms associated with membrane transport, immune/inflammatory signaling, and renal/urogenital development. This was in contrast to cadmium, where proteostasis and protein quality control were the dominant themes. Vanadium and uranium had limited overlaps with either arsenic or cadmium or one another. Response to vanadium revealed solute transport and stimulus detection as well as cell cycle/mitotic regulation and chromosome segregation. Uranium exposure was associated with RNA processing/splicing, proteostasis/ER stress and apoptotic signals, but there were fewer DEGs to define GO terms and themes. The results demonstrate that arsenic and cadmium initiate robust and partially overlapping transcriptional responses as early as 6 h, reflecting shared mechanisms of metal toxicity alongside individual metal-specific signatures. In contrast, uranium and vanadium elicit minimal early (6 h) transcriptional responses but develop more robust signatures by 24 h. These findings demonstrate that environmentally relevant metals act through both shared mechanistic pathways and distinct, metal-specific mechanisms. Understanding their individual and overlapping transcriptional mechanisms is critical for interpreting health risks associated with human exposure.

## 1. Introduction

Humans are exposed to various metals and metalloids with established toxic and carcinogenic potential. For some metals, drinking water maximum contaminant levels (MCLs) have been established by the United States Environmental Protection Agency (USEPA), including antimony, arsenic, barium, beryllium, cadmium, chromium, mercury, selenium, thallium, and uranium [1]. However, exceedance of MCLs for one or more metals may be found in community water systems or in unregulated private wells [2,3,4]. Based on a National Health and Nutrition Examination Survey (NHANES) study, nearly 50% of the United States population is exposed to three or more metals as measured by urinary or blood metal levels [5]. A meta-analysis reported associations with mortality from all causes, cardiovascular disease and cancer, and exposure to metals such as cadmium, lead, and arsenic [6]. Additional studies also link metal exposures to cancer, cardiovascular disease and other adverse human health effects [7,8,9,10].

Metal toxicity is often observed in tissues and organs representing sites of exposure, accumulation, or excretion. Many metals are subject to renal excretion, making the kidney a target for metal toxicity; the type and extent of damage in the kidneys will depend on the form of metal, dosage and duration of exposure [11,12,13,14,15,16]. Many of the USEPA-regulated metals, including arsenic, cadmium, and uranium, exhibit renal toxicity, and the kidney is a site of metal accumulation [13,14,15,16].

Although many metals share underlying mechanisms of toxicity, such as generation of reactive oxygen species leading to oxidative stress and damage to cellular components, metals also differ in specific toxic profiles [9,17,18,19,20]. Arsenic, cadmium, uranium and vanadium are all nephrotoxins [13,14,15,16,21,22,23,24], and evidence for metal co-occurrence is found in the analysis of water and soils in mining areas. For example, elevated concentrations of uranium and vanadium co-occurring as carnotite were found in abandoned mine waste in northeast Arizona [25,26]. Cadmium and uranium levels above the NHANES values for the US population were found in urine samples of pregnant women in the Navajo Nation [27]. Although the EPA has not yet established an MCL for vanadium, arsenic and uranium concentrations above MCLs are found in unregulated water sources in the Navajo Nation, an area of extensive mining activity [2]. These findings and others provide evidence for human exposure to multiple metals.

Despite evidence for metal co-exposures in human populations [2,4,5,27,28], few experimental studies directly compare individual metals in a shared target tissue for toxicity. Renal proximal tubule epithelial (RPTE) cells are vulnerable to heavy metal toxicity, and damage to RPTE cells contributes to renal dysfunction [29,30,31]. The aim of this study was to define gene expression profiles of the nephrotoxic metals arsenic, cadmium, uranium, and vanadium in cultured human primary RPTE cells by RNA sequencing. The principal innovation of this study is the application of a comparative framework to four environmentally relevant metals in the same human renal cell model, aiming to provide mechanistic insights into metal toxicity by identifying shared and distinct cellular responses in this important toxicological target.

## 2. Materials and Methods

### 2.1. Cell Model, Culture Conditions and Metal Treatment

Human renal proximal tubule epithelial (RPTE) cells were purchased from Lifeline Cell Technology (Frederick, MD, USA). Cells used for RNA sequencing were derived from Donor 1 (2-year-old male; Catalog # FC-0013, Lot # 08484). RPTE cell stocks were maintained by plating at 5000 cells/cm^2^ in RenaLife™ Epithelial Medium Complete Kit from Lifeline Cell Technology and splitting weekly, up to the recommended 15 doubling times recommended by the vendor. Cells were grown at 37 °C in 5% CO_2_ in a humidified incubator.

For experiments of metal exposures, RPTE cells were plated at 10,000 cells/cm^2^ and allowed to adhere overnight. Cells were then treated with metals for 6 h or 24 h in triplicate. Metals used for treatment were sodium arsenite (AsNaO_2_) (Sigma-Aldrich, St. Louis, MO, USA, Catalog #71287), uranyl acetate (UO_2_(CH_3_COO)_2_·2H_2_O) (EMS, Morgantown, PA, USA, Catalog #22400), cadmium chloride (CdCl_2_) (Alfa Aesar, Waltham, MA, USA, Catalog #36629) and sodium orthovanadate (Na_3_VO_4_) (Sigma-Aldrich, Catalog #204862). Primary treatments consisted of 10 µM sodium arsenite, 10 µM cadmium chloride, 10 µM sodium orthovanadate, and 30 µM uranyl acetate. These concentrations were selected to maintain at least 70% cell survival after 24 h of exposure (Figure S1). For additional comparison at 24 h, lower-dose treatments were also evaluated using 1 µM sodium arsenite, 1 µM cadmium chloride, 1 µM sodium orthovanadate, and 3 µM uranyl acetate. For collection, cells were washed one time in 1× PBS (Sigma-Aldrich, Catalog #8537) and both timepoints were collected simultaneously using the Qiagen (Valencia, CA, USA) RNeasy Mini Kit (Catalog #217004) with QIAshredders (Catalog #79654) and the RNase-Free DNase Set (Catalog #79254) according to the manufacturer’s protocol. Nucleic acid quantitation and purity were assessed using a NanoDrop ND 1000 Spectrophotometer (Thermo Fisher Scientific, Waltham, MA, USA).

### 2.2. RNA Sequencing

A minimum of 0.5 μg of RNA from each sample was sent to Novogene Corporation Inc. (Sacramento, CA, USA) for quality control analysis and RNA sequencing. Novogene analyzed all samples for RNA quantitation and purity using a NanoDrop Spectrophotometer (Thermo Fisher Scientific) and for RNA integrity using an Agilent 2100 Bioanalyzer (Santa Clara, CA, USA). Only samples with an RIN number ≥ 6.3, a A260/280 ≥ 2.0, and a A260/230 ≥ 2.0 were used for further analysis. Novogene prepared an mRNA library with poly A enrichment for each sample. Sequencing was performed on the Illumina NovaSeq platform using paired-end 150 bp sequencing. Data output included a minimum of 6 Gb/sample with ≥20 million read pairs per sample and a Q30 ≥ 85%. Library quality control was also performed by Novogene to filter out low-quality reads or reads containing adapters, and statistical analysis of data production and quality was also performed. Reads were mapped to the human reference genome assembly GRCh38/hg38.

### 2.3. RNA Sequencing Data Analysis

The RNA-seq data were analyzed by Novogene. Reads were mapped to the human reference genome assembly GRCh38/hg38. The reference genome index was constructed by Hisat2 v2.0.5, and paired-end clean reads were aligned to this reference using Hisat2 v2.0.5. FeatureCounts v1.2.0-p3 was used to count the read numbers mapped to each gene. Next, the expected number of fragments per kilobase of transcript sequence per millions base pairs sequenced (FPKM) of each gene was calculated according to the gene length and number of reads mapped to the gene. A differential expression analysis of two conditions or groups (three biological replicates per condition) was performed using the R package (1.20.0). DESeq, an R package, was used to determine differential expression in a set of digital gene expression data using a statistical model based on the negative binomial distribution. The resulting *p* values were adjusted using the Benjamini–Hochberg approach to controlling the false discovery rate. Genes identified by DESeq with an adjusted *p* value (Padj) < 0.05 were assigned as differentially expressed.

To validate DEGs identified in the transcriptomic analysis, a subset of genes was selected for quantitative PCR (qPCR) analysis based on their distinct expression patterns across metal treatments (arsenic, cadmium, vanadium and uranium) in two additional donors (Figure S2).

### 2.4. Pathway Enrichment Analysis

Gene Ontology (GO) enrichment analysis of DEGs was performed using the clusterProfiler R package (Novogene), which accounts for gene length bias, with Benjamini–Hochberg-adjusted *p* values < 0.05 considered significant. Additional analyses and visualization of enriched GO terms were conducted using NovoMagic™ (Novogene, Beijing, China), a cloud-based platform (version of January 2026) hosting the original project data. Venn diagrams comparing DEG sets were generated from NovoMagic™ exports and visualized using Visual Paradigm Online (version of January 2026).

GO enrichment results were summarized to facilitate biological interpretation and reduce redundancy using REVIGO [32]. REVIGO was applied to the full set of significantly enriched GO terms and performed separately for analyses based on all significant DEGs and on the subset meeting the |fold change| ≥ 2 threshold. REVIGO uses semantic similarity-based clustering to group related GO terms into higher-level biological themes, selects a representative GO term to label each cluster, and assigns semantically related terms as cluster members; cluster size was reported as the number of GO terms assigned to each representative. Cluster representatives were prioritized using low dispensability values and interpreted according to shared biological themes. To assess the impact of high-magnitude expression changes on pathway enrichment, GO analysis was repeated using only DEGs with |fold change| ≥ 2 (|FC| ≥ 2). The top-ranked GO terms (lowest q-values) are shown in scatter-plot figures, with associated genes listed in the corresponding supplemental tables. REVIGO output files were subsequently organized into cluster-level summary tables using an AI-assisted workflow for descriptive synthesis and supplemental reporting; all enrichment analyses and statistical testing were completed prior to this summarization step.

## 3. Results

### 3.1. Transcriptional Response Comparison After 6 h Metal Exposure

There were notable differences between metal exposures for 6 h, as illustrated in the principal component analysis (PCA) (Figure S3). The gene expression profiles of the untreated control and uranium- and vanadium-treated samples cluster closely on both PC1 and PC2, indicating similarity. In contrast, arsenic- and cadmium-treated samples are aligned along PC1 but diverge along PC2, suggesting shared patterns with distinct secondary variation. The total number of DEGs and those with a fold-change greater than 2 or less than −2 (|FC| ≥ 2) are listed in Table 1. Total DEGs for arsenic and cadmium treatment were approximately evenly divided between up- and downregulated genes when all DEGs were considered, whereas the majority of DEGs for vanadium treatment were upregulated genes, and there were no DEGs detected for uranium exposure. When differentially expressed genes were filtered using the |FC| ≥ 2 threshold to focus on transcriptional responses with greater biological relevance, arsenic and cadmium exposure resulted in approximately two and ten times as many upregulated genes as downregulated ones, respectively. All 19 DEGs for vanadium meeting the |FC| ≥ 2 threshold displayed increased transcriptional response. This observation suggests distinct biological responses by each metal.

#### 3.1.1. GO Pathway Analysis of All DEGs After 6 h Exposure

**Figure 1:**
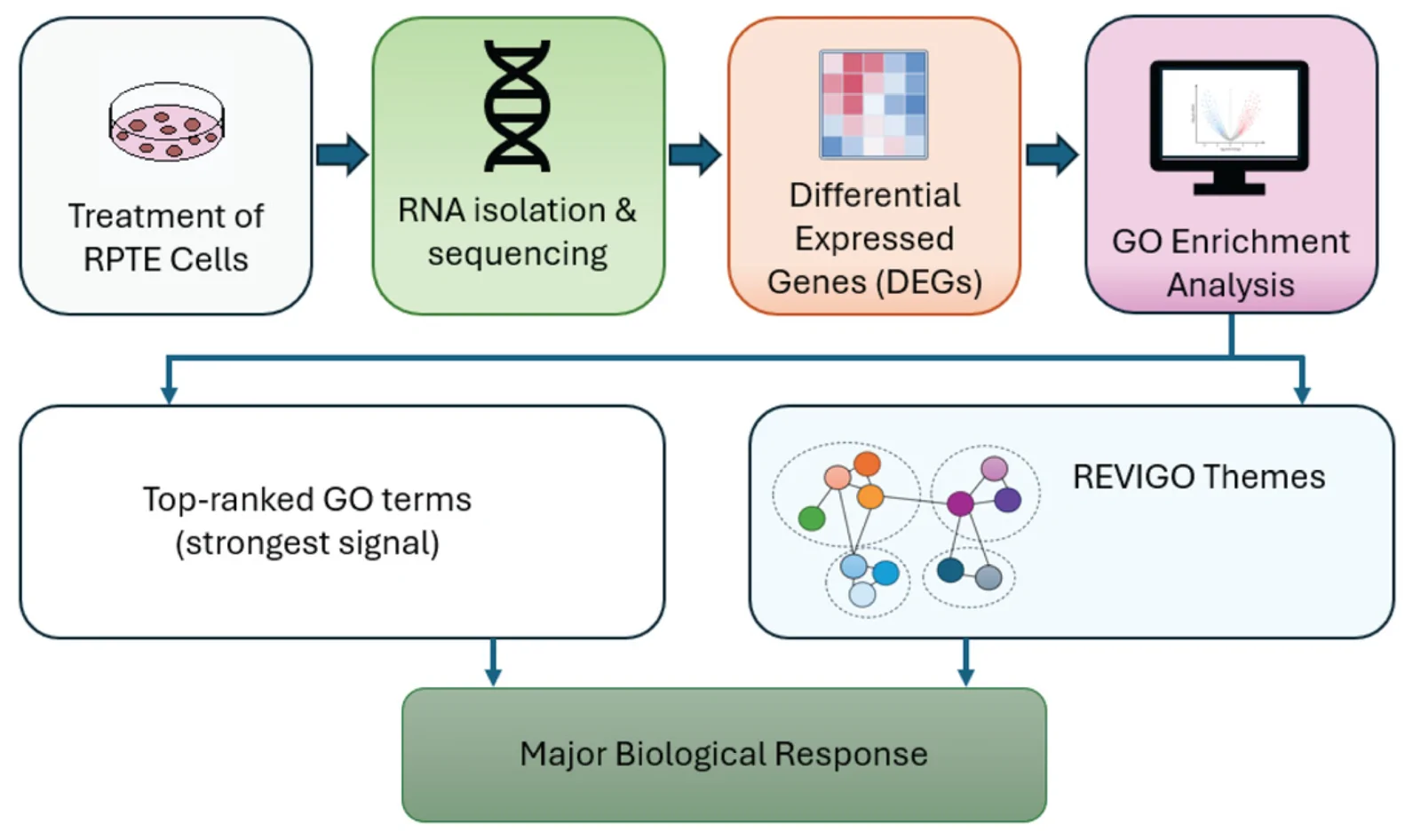
Workflow to identify and interpret biological response themes from RNA-sequencing data. RPTE cells were exposed to treatment conditions, followed by RNA isolation and RNA sequencing. DEGs were identified and analyzed for GO enrichment. Enriched GO terms were then evaluated in two complementary ways: top-ranked GO terms were used to identify the strongest statistical enrichment signals, while REVIGO was used to reduce redundancy and group-related GO terms into broader biological themes. Together, these outputs were used to define the major biological responses associated with each treatment.

GO pathway analysis was performed on all DEGs identified after 6 h exposure to 10 µM arsenic, cadmium, and vanadium or 30 µM uranium. The overall analysis workflow used to move from treatment-specific RNA-seq data to biologically interpretable response themes is summarized in Figure 1.

Table 2 summarizes major REVIGO themes and top-ranked GO terms identified after exposure to each metal for six hours using all DEGs and DEGs with |FC| ≥ 2. The corresponding detailed information listed under supplemental materials is indicated in the Table.

At 6 h, REVIGO clustering following 6 h treatment with 10 µM arsenic indicated renal development and patterning, immune and inflammatory signaling, and cell cycle and mitotic regulation, including chromosome segregation as the major enriched biological themes. The most highly enriched GO terms additionally emphasized regulation of the mitotic cell cycle and G2/M transition, together with autophagy, macroautophagy, proteostasis, and ER-stress responses. Analysis of cadmium-responsive DEGs identified cell cycle checkpoint signaling and cytokinesis as prominent enriched processes. Renal development and patterning, along with hormone- and receptor-mediated signaling, were also among the major biological themes. In contrast, the top-ranked GO terms were concentrated in smaller stress-related themes, including heat and temperature stress, apoptosis, autophagy, and proteostasis/ER stress, rather than in the largest REVIGO clusters.

Together, these results suggest that cadmium exposure at 6 h engages broad regulation of proliferative and developmental programs while also eliciting high-confidence activation of stress-adaptive and cytotoxic pathways consistent with early cellular stress and injury responses. GO pathway analysis did not yield any significantly enriched terms for the 60 DEGs identified after 6 h exposure to 10 µM vanadium, and no DEGs were detected following 6 h treatment with 30 µM uranium, preventing downstream pathway enrichment analysis.

#### 3.1.2. GO Pathway Analysis of DEGs |FC| ≥ 2 at 6 h Metal Treatment

To evaluate GO terms driven by larger-magnitude transcriptional changes following metal exposure for 6 h, GO pathway analysis was conducted for DEGs that were |FC| ≥ 2. REVIGO analysis of arsenic-responsive DEGs with |FC| ≥ 2 emphasized renal and embryonic developmental programs, together with metal response and detoxification themes. Additional themes reflected metal-ion transport, exocytosis, and leukocyte migration, while the top-ranked GO terms highlighted urogenital and skeletal development, heat response, and negative regulation of growth. High fold-change filtering in the arsenic dataset reduced the prominence of the cell cycle/mitosis theme, suggesting that many of those cell cycle-related GO terms were supported primarily by coordinated, lower-magnitude expression shifts rather than large-effect DEGs; in contrast, restricting the analysis to |FC| ≥ 2 increased the relative contribution and ranking of developmental/patterning clusters and made metal response/detoxification signals more apparent.

For cadmium, the major biological themes were focused on metal response/detoxification as well as proteostasis/ER stress. A molecular function theme of neurotransmitter transmembrane transporter activity was driven by solute carrier genes (SLC7A11, SLC22A1, and several SLC6 family transporters), consistent with stress-associated modulation of redox and Na^+^/Cl^−^-coupled solute transport. Most of the top-ranked GO terms were concentrated within these few dominant biological themes of metal response and proteostasis/ER stress. Applying the |FC| ≥ 2 filter for cadmium similarly shifted the enrichment profile toward core stress programs: metal response/detoxification and proteostasis/ER stress rose in relative prominence, while cell cycle/mitosis and development/patterning themes were de-emphasized and no longer among the dominant themes.

#### 3.1.3. Analysis of Overlapping and Unique Enriched Pathways After 6 h Metal Exposure Using DEGs |FC| ≥ 2

**Figure 2:**
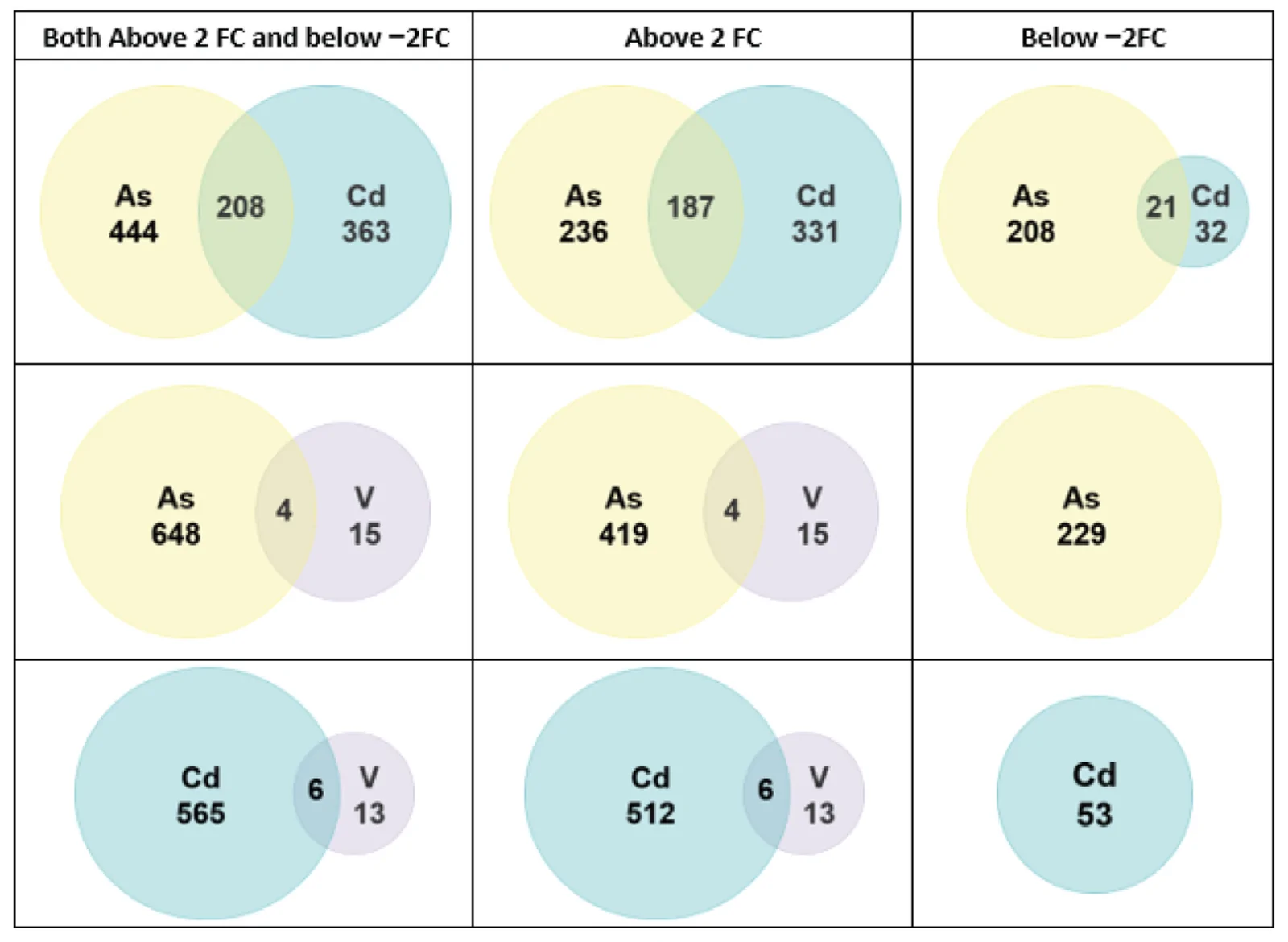
Venn diagrams showing |FC| ≥ 2 DEGs following 6 h metal exposure. Venn diagrams illustrate the overlap of DEGs that met the |FC| ≥ 2 threshold relative to controls identified after 6 h treatment with arsenic (As), cadmium (Cd), and vanadium (V). Each panel compares two metals: As vs. Cd, As vs. V, and Cd vs. V. The overlapping regions indicate genes differentially expressed and in common between the two treatments, whereas the non-overlapping areas represent genes uniquely responsive to the indicated metal pairing.

To further identify metal-specific transcriptional signatures, Venn diagrams were generated using DEGs meeting the |FC| ≥ 2 threshold for arsenic, cadmium, and vanadium (Figure 2). There were no DEGs that met the |FC| ≥ 2 threshold for uranium. Genes unique to each metal and those shared between metal pairs were extracted, and GO enrichment analyses were performed for each resulting gene set.

#### 3.1.4. Analysis of Shared and Metal-Specific Pathways Following 6 h Metal Exposure Using DEGs |FC| ≥ 2

REVIGO analysis of |FC| ≥ 2 DEGs that overlap between arsenic and cadmium revealed functional themes centered on metal response/detoxification. Two themes of proteostasis/ER stress represented by response to unfolded protein and protein folding were enriched. Additionally, the theme heat and temperature stress responses were also enriched, along with extrinsic apoptotic signaling pathways. The top-ranked GO terms were largely captured within the dominant REVIGO clusters, indicating that the enrichment signal is concentrated in a small number of coherent biological programs (Tables S9 and S10). Together, these results indicate that arsenic and cadmium share a transcriptional response characterized by coordinated engagement of proteostasis, metal detoxification, and generalized stress-adaptive pathways.

Metal-specific responses were analyzed using the same approach after excluding DEGs shared between treatments. The distinct arsenic-specific transcriptional response was still dominated by renal/urogenital developmental processes and skeletal development. After removing the shared DEGs dominated by metal detoxification, the enrichment profile shifted toward additional themes, including cell fate determination and terms related to digestive tract development/renal tubule morphogenesis, which further reinforced the broader developmental/patterning response (Tables S11 and S12).

The cadmium-specific response shared broad functional themes with the arsenic and cadmium overlap (e.g., metal handling and proteostasis), but the underlying gene members contributing to these terms differed. In the cadmium-only set, metal response and detoxification themes were supported by a distinct subset of metallothionein-family transcripts (MT1HL1, MT3, MT1A, MT1B). Proteostasis/ER stress signatures were driven primarily by ER quality-control pathways, including ER-associated degradation (ERAD) pathways and ER unfolded protein response (UPR) regulation (Tables S13 and S14).

Together, these results indicate that arsenic and cadmium share coordinated activation of proteostasis, metal detoxification, and generalized stress-adaptive pathways, with cadmium exhibiting metal-specific variations within these shared themes, whereas arsenic exposure continues to modulate developmental processes.

#### 3.1.5. Analysis of Vanadium Compared to Arsenic or Cadmium Using DEGs |FC| ≥ 2 at 6 h

Vanadium exposure elicited a limited transcriptional response at 6 h compared to arsenic or cadmium. Treatment with 10 µM vanadium resulted in 60 DEGs, of which 19 met the |FC| ≥ 2 threshold; however, GO pathway enrichment analysis did not identify any significantly enriched terms for either gene set. Direct comparison of vanadium-responsive genes with arsenic- and cadmium-responsive DEGs revealed a small number of overlapping genes (Table S15) but no coherent enrichment of shared functional pathways. Vanadium-specific DEGs were largely distinct and did not cluster into recognizable processes. Together, these results indicate that 6 h treatment with 10 µM vanadium induces a modest and fragmented transcriptional response that lacks the coordinated pathway-level engagement observed for arsenic and cadmium.

#### 3.1.6. Six-Hour Treatment Summary

When enrichment was performed using all significant DEGs, both arsenic and cadmium showed clustering of themes related to cell cycle/mitotic regulation and developmental/patterning programs. However, arsenic enrichment was dominated by terms reflecting mitotic control, whereas cadmium showed stronger enrichment of apoptosis- and stress-associated pathways. In both treatments, renal development/patterning signals were apparent (e.g., kidney development-related terms), and the highest-ranked enrichment signals also highlighted activation of protein quality-control and autophagy programs consistent with early stress adaptation, along with enrichment of cell fate-related processes.

At high fold-change thresholds, arsenic and cadmium produced overlapping GO enrichment related to cellular stress, damage control, and survival. Cadmium-specific DEGs contributed to many of the same broad themes as the shared response, including metal handling and proteostasis, although the underlying genes differed. In contrast, arsenic-specific DEGs were enriched for distinct developmental and differentiation-related pathways. These findings suggest that cadmium activates related stress-response pathways through a partly distinct set of genes, whereas arsenic produces additional transcriptional changes outside the shared stress response.

### 3.2. Transcriptional Response Comparison After 24 h Metal Exposure

PCA plot of 24 h metal exposure (Figure S4) shows that untreated and uranium- and vanadium-treated cells are clustered on the PC1 and PC2, indicating similarities for these metals. Arsenic and cadmium are similar for PC1 but diverge on PC2, indicating differences in those metals from the group and each other. The total number of DEGs for arsenic, cadmium and vanadium after 24 h treatment had similar proportions of up- and downregulated genes (Table 3). Substantially fewer DEGs were detected in response to uranium, with the majority representing downregulated transcripts. When considering DEGs that met the |FC| ≥ 2 threshold, a greater proportion of arsenic-responsive genes were downregulated, whereas the cadmium and vanadium response was predominantly characterized by upregulated genes.

#### 3.2.1. GO Pathway Analysis of All DEGs After 24 h Exposure

Table 4 summarizes major REVIGO themes and top-ranked GO terms identified after exposure to each metal for 24 h using all DEGs and DEGs with |FC| ≥ 2. The corresponding detailed information listed under supplemental materials is indicated in the Table.

At 24 h, arsenic exposure enriched DEGs that clustered into themes associated with cell cycle regulation, DNA damage, protein polymerization, and intracellular transport/trafficking. The top GO terms additionally emphasized immune and inflammatory activation, including antigen processing and neutrophil degranulation, together with autophagy and protein turnover. In contrast to the 6 h arsenic response, which was characterized by developmental and cell cycle/regulatory pathway enrichment, the 24 h transcriptional profile was dominated by immune activation, cellular trafficking and proteostasis-related processes, indicating a temporal shift from early regulatory adaptation toward sustained stress and inflammatory signaling.

Cadmium exposure was dominated by cell cycle and mitotic processes, including DNA replication and repair, cytokinesis, microtubule regulation, and chromosome segregation. Top-ranked GO terms also highlighted proteostasis and ER-stress responses, particularly responses to unfolded proteins and endoplasmic reticulum stress. Both 6 h and 24 h cadmium responses were characterized by cell cycle, DNA damage, and proteostasis/ER stress; differences were primarily in emphasis, with 24 h showing less enrichment of developmental/patterning and more pronounced enrichment of mitotic progression and replication-associated terms and 6 h showing stronger representation of autophagy/apoptosis-associated stress pathways.

Vanadium exposure was characterized primarily by cell cycle and mitotic regulation, including chromosome segregation, checkpoint signaling, centromeric processes, and nuclear division. RNA processing and mRNA splicing also represented a prominent response. Compared with arsenic and cadmium at 24 h, vanadium exposure showed enrichment of RNA processing terms in addition to cell cycle-associated pathways similar to arsenic and cadmium. Vanadium exposure lacked the antigen presentation and neutrophil-associated immune terms prominent for arsenic or the proteostasis/ER stress terms associated with cadmium.

Uranium exposure was characterized by RNA processing and splicing, proteostasis and ER stress, and intrinsic apoptotic signaling. Regeneration-associated terms were also identified, suggesting activation of broader tissue-repair programs. Because relatively few DEGs were detected, most top-ranked GO terms were captured within the REVIGO themes or remained as individual unclustered terms. Compared with arsenic and cadmium at 24 h, uranium exposure exhibited an enrichment profile that, like vanadium, was dominated by RNA processing/splicing pathways, but uranium included additional enrichment of apoptosis and repair/regeneration terms.

#### 3.2.2. GO Pathway Analysis of DEGs |FC| ≥ 2 at 24 h

At 24 h, arsenic responsive DEGs meeting the |FC| ≥ 2 threshold revealed passive transmembrane transporter activity and regulation of vesicle-mediated transport, including regulation of metal ion transport, as prominent themes. An immune/inflammatory signaling theme was represented by leukocyte migration and monocyte chemotaxis. Additional themes included regulation of nervous system processes, which likely reflect neuroactive/vasoactive control of smooth muscle contraction. The GO term heart contraction reflected a broader theme of vascular tone and contractile regulation, supported by genes involved in vasoactive signaling, calcium handling, and smooth muscle function. The top GO terms focus on ion homeostasis processes (intracellular calcium ion homeostasis), chemokine signaling (leukocyte chemotaxis), and inflammatory response terms (response to lipopolysaccharide). Several highly enriched terms also mapped to signaling and tissue remodeling programs, including the ERK1/2 cascade and its regulation, and extracellular matrix pathways, such as extracellular matrix organization and collagen catabolic process. Compared with the 6 h |FC| ≥ 2 arsenic response, which emphasized developmental/patterning programs with proteostasis/ER stress, the 24 h |FC| ≥ 2 profile shifted toward calcium/divalent cation homeostasis, immune cell migration/inflammatory signaling, metal response/detoxification and extracellular matrix remodeling.

Cadmium-responsive DEGs with |FC| ≥ 2 were broadly associated with metal response and detoxification, including responses to copper, zinc, and other metal ions. Proteostasis and ER-stress pathways were also prominent, with enrichment of the unfolded protein response, protein folding and refolding, and heat-stress processes, together with regulation of sodium, zinc, and calcium ion homeostasis. The most highly enriched GO terms primarily emphasized stress responses to metal ions and cellular responses to unfolded proteins, consistent with activation of metal-handling, protein quality-control, and general stress-response pathways. The 24 h cadmium |FC| ≥ 2 enrichment profile closely mirrors the 6 h |FC| ≥ 2 response, with dominant themes centered on metal ion homeostasis/detoxification and proteostasis/ER stress.

Vanadium-responsive DEGs with |FC| ≥ 2 were associated with solute, anion, and organic acid transport. Channel and transmembrane transporter functions were therefore the dominant patterns identified in the REVIGO analysis. The most highly enriched GO terms further emphasized calcium ion homeostasis and metal-ion transport, while additional individual terms reflected stimulus detection, extracellular matrix assembly, and muscle contraction. In contrast to arsenic and cadmium at 24 h, which were characterized by immune signaling and metal detoxification programs respectively, high-magnitude vanadium-responsive gene expression changes preferentially map to ion transport, membrane transport, and circulation regulation pathways.

HSPA6 was the only DEG that met the |FC| ≥ 2 threshold for 30 µM UA 24 h treatment.

#### 3.2.3. Analysis of Shared and Metal-Specific Pathways Following 24 h Metal Exposure Using DEGs |FC| ≥ 2

**Figure 3:**
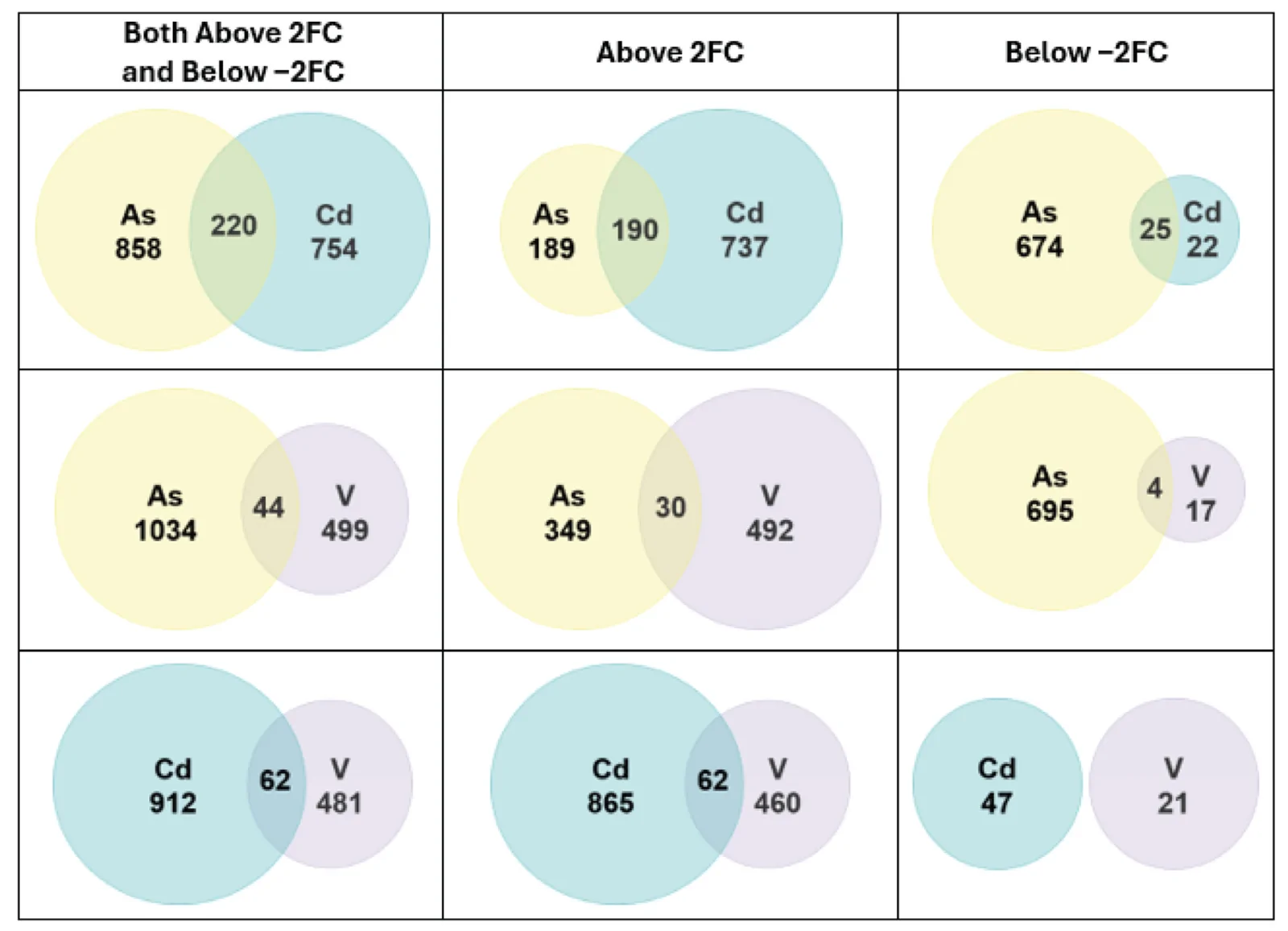
Comparison of DEGs |FC| ≥ 2 between metals after 24 h treatment. Venn diagrams comparing the DEGs of cells treated with 10 µM arsenic, 10 µM cadmium or 10 µM vanadium compared to the no-treatment control. No Venn diagrams were created for combinations with uranium because only one gene (HSP6A1) met the |FC| ≥ 2 threshold.

To further identify metal-specific transcriptional signatures, Venn diagrams were generated using DEGs meeting the |FC| ≥ 2 for arsenic, cadmium, and vanadium (Figure 3). Genes unique to each metal and those shared between metal pairs were extracted, and GO enrichment analyses were performed for each resulting gene set.

REVIGO analysis of the overlapping arsenic–cadmium DEGs at 24 h identified three main themes: metal response and detoxification, immune and inflammatory signaling, and proteostasis/ER stress. The top-ranked GO terms were primarily related to metal handling and homeostasis (Tables S30 and S31). These findings indicate that the shared high-magnitude 24 h response to arsenic and cadmium converges on core metal stress-adaptive programs, with smaller accompanying signals related to proteostasis/ER stress and immune signaling.

REVIGO clustering of the arsenic-specific 24 h |FC| ≥ 2 DEG set, after excluding DEGs shared with cadmium, produced a cluster profile that closely matched the original arsenic 24 h |FC| ≥ 2 enrichment results (Tables S32 and S33). In the cadmium-only |FC| ≥ 2 set, the enriched GO term list was small, and REVIGO retained most terms as single-term representatives (i.e., cluster size = 1) matching the top-ranked GO terms. The individual terms consistently mapped to proteostasis/protein quality-control themes (Tables S34 and S35).

The small arsenic–vanadium overlap |FC| ≥ 2 DEG produced limited GO enrichment, mainly related to neuronal development and regulation of blood circulation. Because these terms were supported by very few DEGs, they likely reflect narrow gene-set effects rather than a strong shared biological response (Tables S36 and S37).

Because the 24 h |FC| ≥ 2 arsenic–vanadium overlap comprised only 44 DEGs and only a few of these mapped to six enriched GO terms, removing the overlap had minimal impact on pathway-level interpretation, and the arsenic- and vanadium-specific REVIGO and the most highly enriched GO terms showed little variation from the original arsenic 24 h |FC| ≥ 2 enrichment profile (Tables S38–S41).

The small cadmium–vanadium overlap |FC| ≥ 2 at 24 h produced no significantly enriched GO terms, indicating little shared functional organization. Removing the shared DEGs had minimal effect on the cadmium-specific results but shifted the vanadium-specific results from broader biological processes to a more specific molecular function process where the focus is on the activity of the calcium transmembrane transporter specifically as opposed to the overall biological process of how calcium ions are transported into a cell (Tables S42–S45). In contrast to the strong metal detoxification signature shared between arsenic and cadmium and the neuronal differentiation signature shared between arsenic and vanadium, cadmium and vanadium did not exhibit a coherent shared high-magnitude transcriptional program at 24 h.

### 3.3. Transcriptional Response Comparison at Lower Dose Metal Exposure for 24 h

A low-dose treatment was included to provide insight into gene expression changes that may occur at more environmentally relevant exposure level. Transcriptional response was tested for each metal at a ten-fold lower dose for 24 h (Table 5). As observed at the higher dose, arsenic and cadmium had the most robust changes in gene expression, with 53 and 27 DEGs, respectively, although the number of DEGS was substantially reduced. Vanadium and uranium treatment resulted in very few (V) and no DEGs (UA).

**Figure 4:**
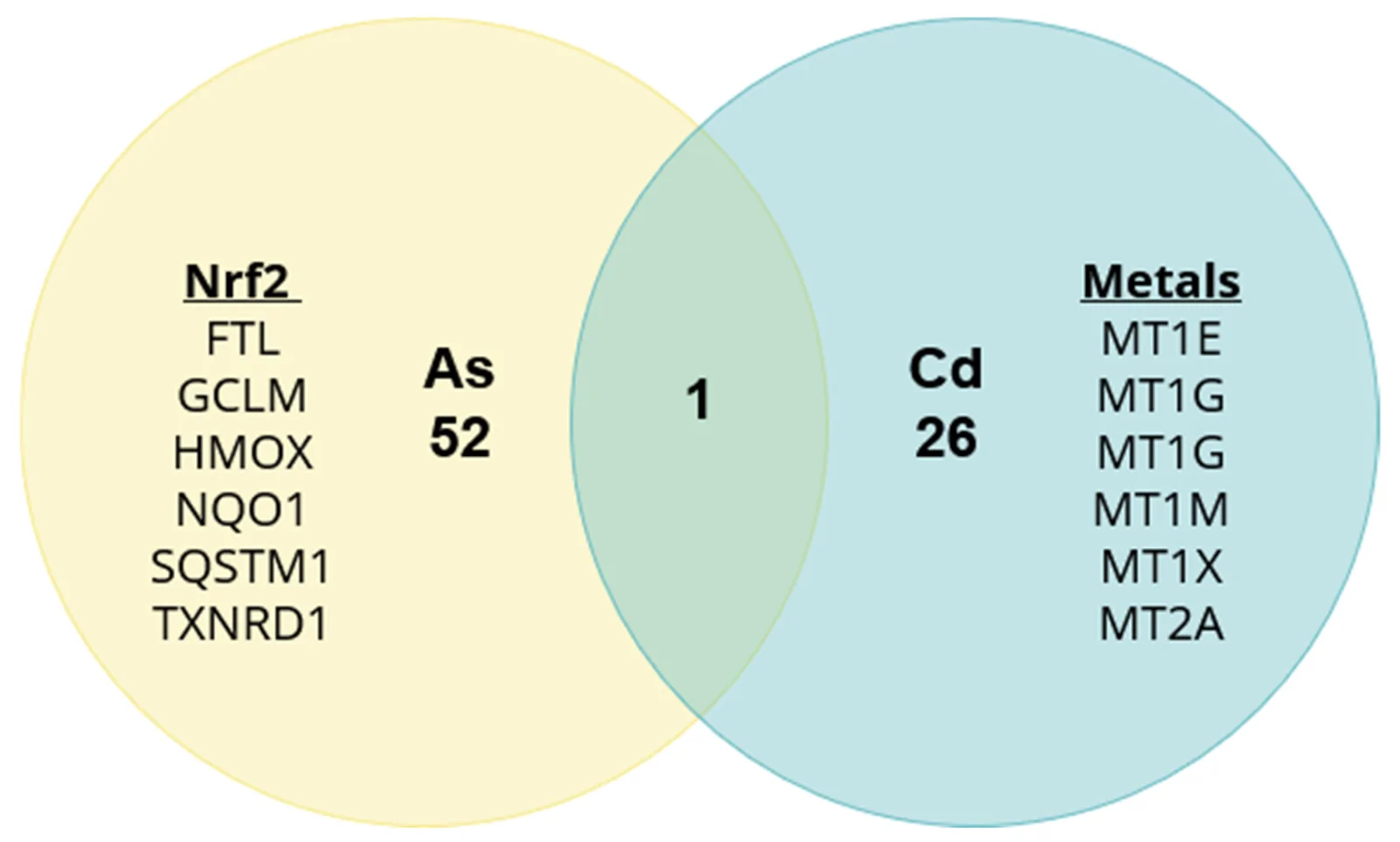
Comparison of DEGs between arsenic and cadmium after 24 h treatment. Venn diagrams comparing the DEGs of cells treated with 1 µM arsenic and 1 µM cadmium compared to no-treatment control. No Venn diagrams were created for vanadium or uranium due to the low number of DEGs.

Many of the DEGs increased in response to 1 µM As treatment for 24 h are associated with the Nrf2 pathway, including FTL, GCLM, HMOX1, NQO1 and SQSTM1 (Figure 4 and Table S46). DEGs that were decreased included solute and ion transporters (SLC5A3, 6A6, 34As and a sodium channel SCNN1A) and the cell cycle regulators CTDSP1 and MCM7. All but four DEGs were present in the low dose but not in the high dose (Table S46). Changes in most genes kept a common trajectory of up or down, with a greater magnitude of change at the higher dose (Table S46). The only enriched GO term for lower dose As treatment was secondary lysosome (GO:0005767) that includes three genes (FTL, FTH1 and SQSTM1).

DEGs increased in response to 1 µM cadmium after 24 h treatment, including genes encoding metal homeostasis and detoxification genes such as metallothioneins (MT1G, X, M, F, E and L) (Table S47). Decreased DEGs included some transcription factors (NR2F6 and ERF) and transporters (SLC16A3). Only 15/27 DEGs in the low dose were also present in the high dose. The majority of DEGs common to low- and high-dose cadmium were upregulated metallothioneins (Table S47). REVIGO themes were focused on metal response/detoxification, with representing GO terms including stress response to metal ions (Table S48). The majority of the top terms fit within this theme (Table S49). Only one gene was shared between the low arsenic and cadmium doses (MALAT1).

The four DEGs identified after 1 µM vanadium 24 h treatment were RAB3B, CD109, CBX5 and LENG8, which produced no enriched GO terms (Table S50). There were no DEGs for lower-dose uranium. Vanadium and arsenic also shared one DEG (RAB3B).

Low-dose arsenic (1 µM, 24 h) preferentially enriched an NRF2-linked oxidative stress signature, and these genes remained induced at 10 µM. There was a distinct difference between DEGs enriched by low-dose arsenic and cadmium. However, metal handling/detoxification terms were detectable in the 10 µM arsenic (24 h) dataset, even though they were not among the dominant REVIGO themes or the top-ranked GO terms in either the all-DEG or |FC| ≥ 2 analyses, suggesting that metal detoxification-related changes were secondary or supported by a smaller/less-coherent gene subset relative to the broader developmental, cell cycle and immune programs maintained by arsenic. In contrast, low-dose cadmium (1 µM, 24 h) primarily enriched metal stress pathways; while these themes were not prominent in the 24 h all-DEG cadmium enrichment, they emerged clearly in the |FC| ≥ 2 analysis, consistent with metal-response genes exerting a high-magnitude effect that drives pathway enrichment when the analysis is restricted to large fold changes.

## 4. Discussion

The toxicity of heavy metals and metalloids is dependent on the chemical properties of a metal and exposure characteristics, such as dose and duration [11]. Organ-specific toxicity may occur when a target organ is a site of exposure (e.g., lung) or excretion (e.g., kidney). Many metals are excreted through the renal route and accumulate in kidney tissues, leading to greater localized exposure and potential for damage. Various mechanisms account for metal-induced toxicity and interference with the antioxidant defense systems, with attendant increases in oxidative damage shared by many metals [8,9,19,33,34]. Other mechanisms include displacement or substitution of essential metals with toxic metals in critical protein binding sites and modulation of intracellular signaling pathways, leading to changes in gene expression and essential cellular processes, as well as other mechanisms [8,11,31,34,35]. In this study, we directly compared the gene expression profiles of four nephrotoxic metals by RNA sequencing to better understand similarities and distinctions between specific metal exposures.

For analysis, we interpreted GO enrichment results using a stepwise strategy designed to capture both the full transcriptomic response and the biological programs most strongly driven by large expression changes (Figure 1). GO enrichment was performed using all significant DEGs and summarized in two complementary ways: the top-ranked GO terms (lowest adjusted *p* values) were reported to highlight the strongest statistical signals, and REVIGO clustering was used to reduce redundancy and organize the complete term list into higher-level biological themes. In order to assess pathway-level interpretation focused on high-magnitude transcriptional effects, enrichment was repeated after restricting DEGs to |FC| ≥ 2, and results were compared with the all-DEG output to identify which themes are retained versus those supported primarily by coordinated lower-magnitude shifts. Finally, Venn diagram-based DEG partitioning (shared vs. metal-specific gene sets) was used to separate convergent stress/metal-handling responses from metal-specific programs; GO enrichment, top-ranked term reporting, and REVIGO summarization were applied to each partition to determine whether shared pathways reflect common toxicant-response mechanisms and whether metal-specific DEGs reveal distinct biological processes and time-dependent progression of response. Together, this layered approach increases transparency, reduces redundancy, and strengthens confidence that reported themes reflect robust biological programs rather than artifacts of term overlap or threshold choice.

RPTE cellular transcriptomic response to arsenic and cadmium was the most similar in terms of rapid and robust gene expression changes based on DEG count (Table 1 and Table 3), PC analysis (Figures S2 and S3), Venn diagrams (Figure 2 and Figure 3) showing shared DEGs and shared functional themes as identified by REVIGO analysis and top GO terms. The two metals shared GO terms and REVIGO themes related to stress response, which was confirmed by qPCR in cultured RPTE cells from three independent donors (Figure S2). This is consistent with evidence in various cell and organ systems for oxidative stress, metallothionein induction and proteotoxic response as underlying mechanisms of metal toxicity [9,11,19,31,34,35]. For both arsenic and cadmium, HMOX1, NQO1 and MTG1 induction was of similar magnitude at 6 h metal treatment and sustained at 24 h, whereas HSPA6 was less pronounced after arsenic treatment at 24 h when compared to cadmium (Figure S2). At a lower treatment dose of 1 µM arsenic or cadmium for 24 h, arsenic-regulated DEGs included the oxidative stress markers HMOX1 and NQO1 and other genes regulated by the nuclear factor erythroid 2-related factor 2 (Nrf2) signaling pathway. In contrast, DEGs associated with low-dose cadmium included eight metallothionein genes represented in metal detoxification GO terms, which were not evident with arsenic treatment, and only one DEG, MALAT1, was common to both metals at the low dose (Figure 4). The numerous metallothionein DEGs are consistent with a previous report of early and persistent activation of metal-responsive transcription factor-1 (MTF1) involved in metallothionein expression by cadmium in induced human renal proximal tubule-like cells [36]. Although both cadmium and arsenic promote expression of transcripts associated with cellular stress, underlying differences in the predominant mechanisms may account for the differential stress transcriptomic responses.

Analysis of the higher dose 2FC data revealed additional unique aspects of arsenic and cadmium exposures. There was greater diversity of arsenic themes, including transmembrane and vesicle-mediated transport, immune and inflammatory signaling, other signaling pathways, extracellular matrix remodeling and genes involved in kidney and urogenital development (Tables S24 and S25). The potential relevance of gene expression related to renal development is suggested by a recent report. Arsenic exposure inhibited growth and branching morphogenesis in mouse kidney embryonic kidney explants and decreased nephron number in vivo [37]. These findings suggest arsenic exposure may contribute to adverse renal effects during development. In contrast, cellular processes unique to cadmium in the 2FC DEG set were dominated by proteostasis and protein quality control (Tables S26 and S27). Mechanistically, cadmium displacement of metals such as calcium, iron and zinc may disrupt vulnerable protein structures and prompt the cellular responses required to address damaged and misfolded proteins in addition to oxidative damage.

In contrast to arsenic and cadmium, vanadium is not classified as a human carcinogen by the International Agency for Research on Cancer (IARC), although it shares nephrotoxic properties with both metals [13,15,16]. There were distinct differences in cellular response to vanadium when compared to arsenic or cadmium as indicated by the PCA plot (Figures S2 and S3). Fewer DEGs were detected in response to vanadium, and the response was delayed with far fewer DEGs at 6 h of treatment vs. 24 h treatment (Table 1 and Table 2). At either timepoint, the number of shared genes between vanadium and cadmium or arsenic was markedly less than that observed for arsenic and cadmium (Figure 2 and Figure 3). The DEGs at the 2FC threshold shared by vanadium and arsenic had a theme of neuronal differentiation and cortical development (Table S36), whereas no enriched GO terms were evident for the genes in common with cadmium. At the lower dose of 1 µM, only four DEGs were detected for vanadium, with a single gene (RAB3A) shared with arsenic at the same dose and time (Table S46).

Despite reports that vanadium promotes oxidative stress, no significant increase was detected for HMOX1 or NQO1 nor the stress response genes MT1G or HSPA6 in RPTE cells from three independent donors (Figure S2). However, vanadium was the only metal that significantly induced MUC20, a mucin highly expressed in human kidney proximal tubules and upregulated upon renal injury [38]. At the 2FC threshold, other genes and processes unique to vanadium were predominantly related to transmembrane transport and channel activity (Tables S28 and S29). The limited number of genes and pathways or themes in common between vanadium and arsenic or cadmium suggests divergence of signaling mechanisms between the metals leading to a vanadium outcome largely related to ion and solute transport. Vanadium is known to inhibit protein tyrosine phosphatases, which leads to modulation of various signaling cascades [39]. The limited similarities in gene expression profiles and pathways for arsenic, cadmium and vanadium suggest that vanadium is acting largely through mechanisms that are not broadly shared by arsenic or cadmium in RPTE cells.

Based on the PCA plots, uranium and vanadium were more similar to one another than either arsenic or cadmium (Figures S3 and S4), and both had fewer DEGs than either arsenic or cadmium (Table 2 and Table 3). There were no DEGs detected after 6 h of uranium treatment nor at the 10-fold lower dose of 3 µM for 24 h. The 92 DEGs at 24 h of treatment provided uranium-enriched GO terms, with dominant functional themes centered on RNA processing/splicing, proteostasis/ER stress (Tables S22 and S23) and terms related to apoptosis. In this regard, uranium shared properties with cadmium to a greater degree than the other metals. The limited number of DEGs could not be attributed to a lack of uranium uptake into cells as measured by ICP-MS (Figure S5). Similar to vanadium, uranium did not increase the oxidative stress genes NQO1 or HMOX1 (Figure S2) or MT1G, but uranium did increase expression of HSPA6, as also detected for arsenic and cadmium. Uranium exposure also did not promote an oxidative stress response in T-lymphocyte cell lines or in an exposed human population [40,41,42] in contrast to arsenic in the same systems. The lack of oxidative stress-induced gene expression was not surprising at the dose of uranyl acetate used in this study. Evidence for oxidative stress is more typically detected at uranium concentrations between 100 µM and 1 mM in cell-based studies [8].

## 5. Conclusions

**Figure 5:**
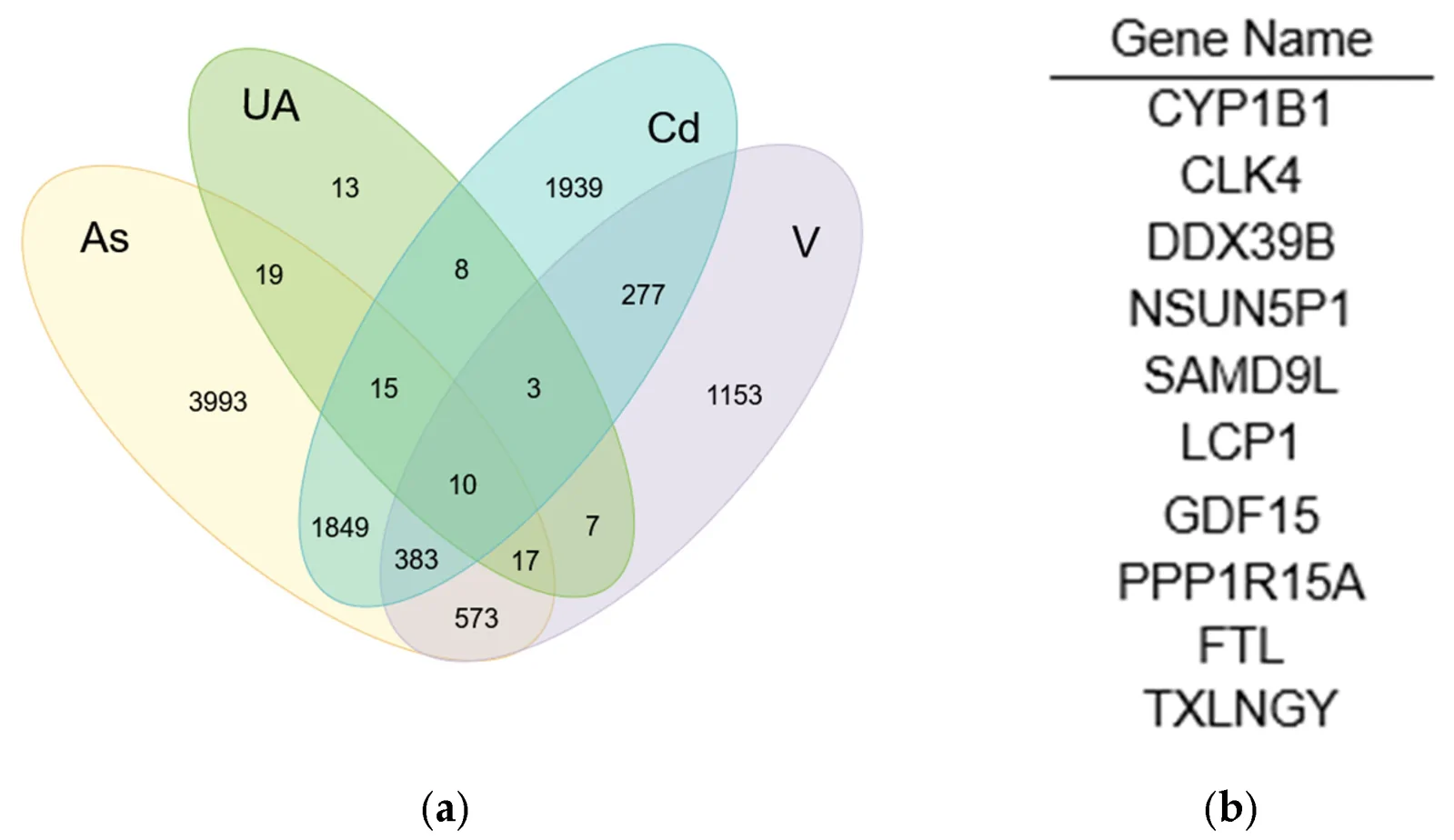
Venn diagram showing overlap of DEGs across metal exposures after 24 h treatment. The number of DEGs shared and unique among arsenic (As), cadmium (Cd), vanadium (V), and uranium (UA) treatments after 24 h treatment (**a**); list of 10 DEGs common to all metal treatments (**b**). Overlapping regions represent genes differentially expressed in more than one exposure condition, whereas non-overlapping regions represent exposure-specific DEGs. Differential expressions were defined using a *p*-adjusted value of 0.05.

Comparisons of cellular response to specific metals across studies are challenging because of differences in cell and tissue models and metal exposure and dosing procedures in the published literature. By directly comparing transcriptomic responses to four known nephrotoxic metals in the highly relevant normal human RPTE cells, we identified similarities and differences between metal pairs as highlighted in the results. The differences in metal response are more striking when all four metals are taken into consideration (Figure 5a). Only ten DEGs were shared in common by arsenic, cadmium, uranium and vanadium (Figure 5b). These genes span metabolism, RNA processing, immune and inflammatory signaling and cytoskeleton and trafficking, but were too few to yield shared GO terms or REVIGO themes. An earlier study in a human bronchial epithelial cell line using a 1200 gene nylon array found only one gene (hsp90) common among four metal treatments (cadmium, chromium, nickel and arsenic) [43]. These findings and those reported herein indicate that there are meaningful differences between metal response in a specific target cell or tissue type.

Limitations to the current study include single metal exposures and analysis of response in a single target cell type for metal toxicity. Environmental exposure to metals by individuals or within a population is generally a metal mixture rather than a single contaminant, and systemic exposures affect multiple tissues and organs beyond those associated with uptake or excretion. The current approach could be applied to other target cells or tissues from in vivo exposures to interrogate tissue-specific vulnerabilities. Although the current studies identify overlapping and metal-specific transcriptional signatures, future research will be needed with metal combinations to better understand how complex environmental exposures may contribute to kidney toxicity. The scope of potential interactions, whether additive, synergistic or antagonistic, is poorly understood, thereby highlighting the need for studies that better reflect real-world exposures. Acknowledging these limitations, the current work illustrates how direct comparisons of metals in biologically relevant models can reveal a more nuanced understanding of the consequences of metal exposure that may be important to further our understanding of toxicity and the potential to mitigate the adverse effects of metals.

## Figures and Tables

**Table 1:** Total number of DEGs and number of DEGs |FC| ≥ 2 after 6 h treatment.

	10 µM As		10 µM Cd		10 µM V		30 µM UA	
	Total	2 FC	Total	2 FC	Total	2 FC	Total	2 FC
**Up**	2651	423	2222	518	51	19	0	0
**Down**	2589	229	1629	53	9	0	0	0
**Total**	5240	652	3851	571	60	19	0	0

**Table 2:** Major REVIGO themes and top-ranked GO terms identified for each metal at 6 h using all DEGs and DEGs with |FC| ≥ 2.

All DEG		|FC| ≥ 2	
**Arsenic**			
REVIGO:	Table S1	REVIGO:	Table S5
Renal development/patterning Immune/inflammatory Cell cycle and mitosis		Renal developmental/patterning Metal response/detoxification Metal ion transport/exocytosis Leukocyte migration/chemotaxis	
Top GO:	Table S2	Top GO:	Table S6
Regulation of mitotic cell cycle/G2/M phase Autophagy/macroautophagy Proteostasis/ER stress		Urogenital, skeletal, renal system development Response to heat Negative regulation of growth	
**Cadmium**			
REVIGO:	Table S3	REVIGO:	Table S7
Cell cycle checkpoints, cytokinesis Renal development/patterning Hormone and receptor-mediated signaling		Metal response/detoxification Proteostasis/ER stress Sodium-dependent transmembrane transport	
Top GO:	Table S4	Top GO:	Table S8
Heat/temperature stress Apoptosis/cell death Autophagy Proteostasis/ER stress		Response to copper, cadmium, zinc ion Response to unfolded protein Proteostasis/ER stress	

The background colors represent the same colors used in the Venn diagrams.

**Table 3:** Total number of DEGs and number of DEGs |FC| ≥ 2 after 24 h treatment.

	10 µM As		10 µM Cd		10 µM V		30 µM UA	
	Total	2 FC	Total	2 FC	Total	2 FC	Total	2 FC
**Up**	3393	379	2670	927	1357	522	23	1
**Down**	3466	699	1814	47	1066	21	69	0
**Total**	6859	1078	4484	974	2423	543	92	1

**Table 4:** Major REVIGO themes and top-ranked GO terms identified for each metal at 24 h using all DEGs and DEGs with |FC| ≥ 2.

All DEG		|FC| ≥ 2	
**Arsenic**			
REVIGO:	Table S16	REVIGO:	Table S24
Regulation of protein polymerization, binding Cytokinesis and DNA damage Intracellular transport/trafficking		Passive transmembrane, vesicle-mediated and metal ion transport Immune/inflammatory Regulation of smooth muscle	
Top GO:	Table S17	Top GO:	Table S25
Antigen processing, neutrophil activation/degranulation Autophagy/protein turnover		Inflammation and immune response Renal development/tissue remodeling Ion homeostasis and cellular signaling	
**Cadmium**			
REVIGO:	Table S18	REVIGO:	Table S26
Cytokinesis and cell cycle progression DNA strand elongation/break repair Microtubule regulation		Metal response/detoxification Proteostasis/ER stress Ion homeostasis (Zn, Ca^2+^)	
Top GO:	Table S19	Top GO:	Table S27
Mitotic regulation/chromosomal segregation ER stress/response to unfolded protein		Stress response to metal ion ER stress/response to unfolded protein	
**Vanadium**			
REVIGO:	Table S20	REVIGO:	Table S28
Cell cycle and mitosis RNA processing/splicing		Solute/anion/organic acid transport Channel activity Transmembrane transporter activity	
Top GO:	Table S21	Top GO:	Table S29
RNA/mRNA splicing Mitotic cell cycle checkpoint signaling/nuclear division		Calcium ion homeostasis Metal ion transport	
**Uranium**			
REVIGO:	Table S22		
RNA processing/splicing Proteostasis/ER stress Apoptosis/cell death, regeneration		Only HSPA6 met the |FC| ≥ 2 criteria	
Top GO:	Table S23		
RNA/mRNA splicing Response to unfolded protein Intrinsic apoptotic signaling			

The background colors represent the same colors used in the Venn diagrams.

**Table 5:** Total number of DEGs after low dose 24 h treatment.

	1 µM As	1 µM Cd	1 µM V	3 µM UA
**Up**	34	16	3	0
**Down**	19	11	1	0
**Total**	53	27	4	0

## Data Availability

RNA-Seq data generated for this study have been deposited in the NCBI Sequence Read Archive under BioProject PRJNA1474480 and SRA submission SUB16218088. The dataset is titled “RNA-Seq dataset for comparative metal toxicity response in primary human renal epithelial cells (6 and 24 h)” and includes 42 RNA-Seq runs with accession numbers SRR38976989–SRR38977030. The SRA records can be accessed through NCBI SRA using the run accession format, for example: https://www.ncbi.nlm.nih.gov/sra/SRR38976989. URL accessed on 20 June 2026. In accordance with the SRA release timeline selected at submission, the data are under temporary embargo and will become publicly available one year after submission, on 1 June 2027. Supporting data (e.g., GO/REVIGO summary tables) are provided in the Supplementary Materials.

## Supplementary Materials

The following supporting information can be downloaded at: https://www.mdpi.com/article/10.3390/toxics14080648/s1, Supplemental Figures S1–S5 (with accompanying legends and supplemental text) are provided in the Supplementary Word document, and Tables S1–S50 are provided in the Supplementary Excel file (including GO enrichment outputs and REVIGO cluster-level summary tables for all analyses described).

## Abbreviations

The following abbreviations are used in this manuscript:

As	Sodium arsenite
Cd	Cadmium chloride
DEG	Differentially expressed gene
ER	Endoplasmic reticulum
|FC| ≥ 2	2-fold change
GO	Gene ontology
hr	Hour
ICP-MS	Inductively coupled plasma mass spectrometry
MCL	Maximum contaminant level
PCA	Principal component analysis
qPCR	Quantitative polymerase chain reaction
RPTE	Renal proximal tubule epithelial
UA	Uranyl acetate
V	Sodium orthovanadate
