# Supplementary material for: Arsenic, Cadmium, Uranium and Vanadium Produce Shared and Distinct Gene Expression Signatures in Primary Human Renal Cells
Source: Toxics. 2026 Jul 23;14(8):648. doi: 10.3390/toxics14080648 (PMC13517258; doi:10.3390/toxics14080648)
Supplement: Supplementary file 1 [file toxics-14-00648-s001.zip › RPTE Supplemental FINAL.pdf]

Figure S1. Viability curves for metal treatment of RPTE cells.

RPTE cells were plated in quadruplicate at a concentration of 5000 cells/cm^2 in 96-well tissue-culture plates (Greiner cat# 655180). Cells were treated with metals 24 hours after initial plating. Treatments for RPTE were as follows: untreated (NT), sodium arsenite (As), uranyl acetate (UA), cadmium chloride (Cd), and sodium orthovanadate (V) 0-300 μM for 24h. Viability was measured using PrestoBlue® (Life Technologies, CA, USA). PrestoBlue® was added to each well at a final volume of 10% according to manufacturers' instructions, then plates were returned to the cell culture incubator for an additional 2 hours. Fluorescence was measured using a microplate reader (SpectraMAx M2e, Molecular Devices) with excitation 555 nm and emission 585 nm. Values were normalized to untreated controls. Doses selected for subsequent studies were based on a minimum of 70% survival after 24hr exposure to metal.

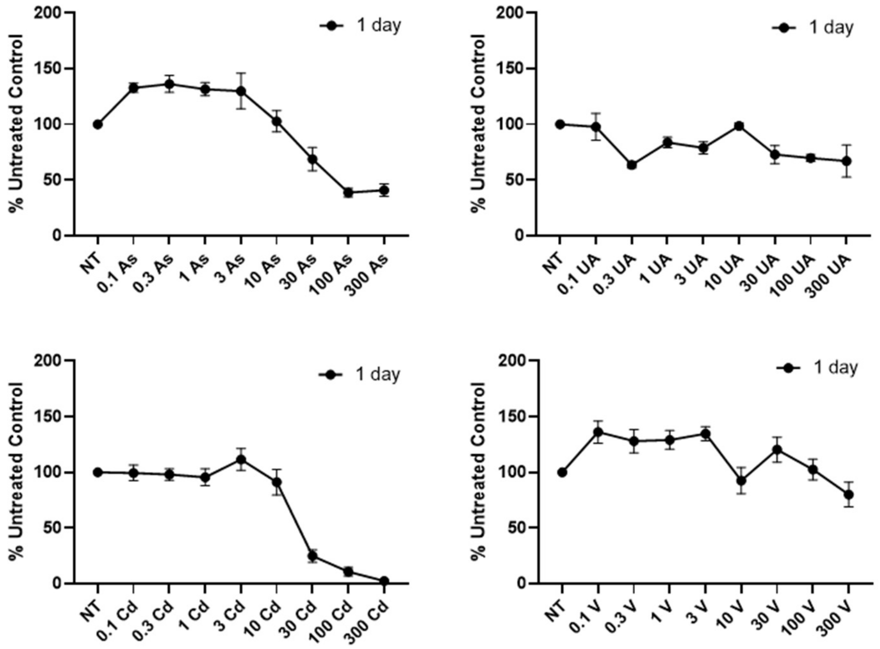

Figure S1. Viability of RPTE cells treated with arsenic, uranium, cadmium or vanadium. RPTE cells were exposed to UA, As, Cd or V for 1 day and viability was measured using PrestoBlue®. All treatment doses are in μM. Viability for doses used in the RNA sequencing studies include 10 μM As 102%, 30 μM UA 73%, 10 μM Cd 91%, 10 μM V 93%. Graphs represent mean ± SEM of at least 3 independent experiments. Reported values are relative to untreated control (NT).

Figure S2. PCR Validation of select genes in additional donors

RPTE cells from three independent donors were obtained from Lifeline Cell Technology. Donor 1 was used for the RNA sequencing analysis (described in section 2.1), Donor 2, a 41-year-old male (Catalog # FC-0013, Lot # 02684) and Donor 3, a 2-month-old female (Catalog # FC-0013, Lot # 00991). Cells from all 3 donors were grown, treated with metals and harvested as described in section 2.1 for PCR validation of RNA sequencing results. The RNA samples were collected using TRIzol (Invitrogen, CA, USA) per manufacturer instructions and quantified using a Nanodrop ONE spectrophotometer (Thermo Fisher Scientific, Waltham, MA). cDNA from all samples was synthesized from 1 µg of RNA using the High-Capacity cDNA Reverse Transcription Kit from Thermo Fisher Scientific (Waltham, MA).

Quantitative real-time (RT-qPCR) was used to examine a subset of relevant genes that were differentially expressed by metal treatment. Eight genes were selected for PCR validation. Genes selected represent mechanisms of metal toxicity including oxidative stress and detoxification (HMOX1, NQO1, HSPA6 and MT1G), cellular processes including autophagy and iron sequestration (SQSTM1 and FTH1) and genes that were increased or decreased in response to some, but not all, the metals (XAF1, MUC20).

RT-PCR was performed in triplicate using SYBR Green PCR primers and SYBR Green Supermix from Bio-Rad (Hercules, California) on the BioRad CFX384 PCR System. Cycling parameters began with an initial denaturation step at 95°C for 30 seconds. 40 amplification cycles were then performed which consisted of denaturation at 95°C for 5 seconds and annealing/elongation at 60°C for 30 seconds. Gene expression analysis was performed using delta-delta Ct with 18S as the reference gene.

PCR Validation Primers

**Gene**	**Forward**	**Reverse**
18S	CGGAGGTTCGAAGACGATCAGATA	TTGGTTTCCCGGAAGCTGCC
HMOX1	TCCTGGCTCAGCCTCAAATG	CGTTAAACACCTCCCTCCCC
NQO1	CGCAGACCTTGTGATATTCCAG	CGTTTCTTCCATCCTTCCAGG
**Gene**	**Proprietary Assay ID**	
HSPA6	qHsaCED0003684	
MTG1	qHsaCED0020789	
FTH1	qHsaCED0038139	
SQSTM1	qHsaCED0045925	
XAF1	qHsaCID0018158	
MUC20	qHsaCED0004608	

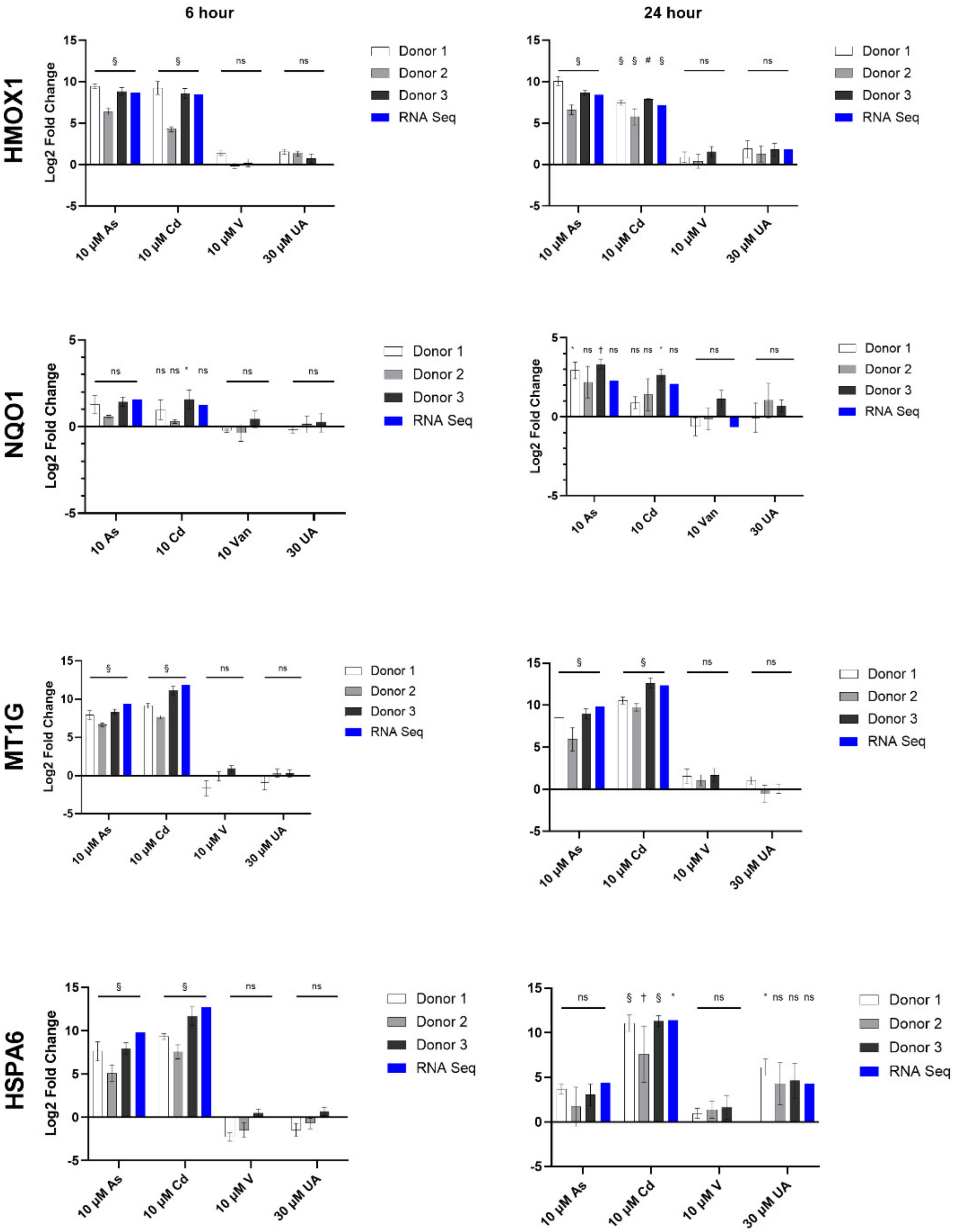
6 hour
24 hour
HMOX1
NQO1
MT1G
HSPA6
Log2 Fold Change
Donor 1
Donor 2
Donor 3
RNA Seq
10 µM As
10 µM Cd
10 µM V
30 µM UA
10 As
10 Cd
10 Van
30 UA

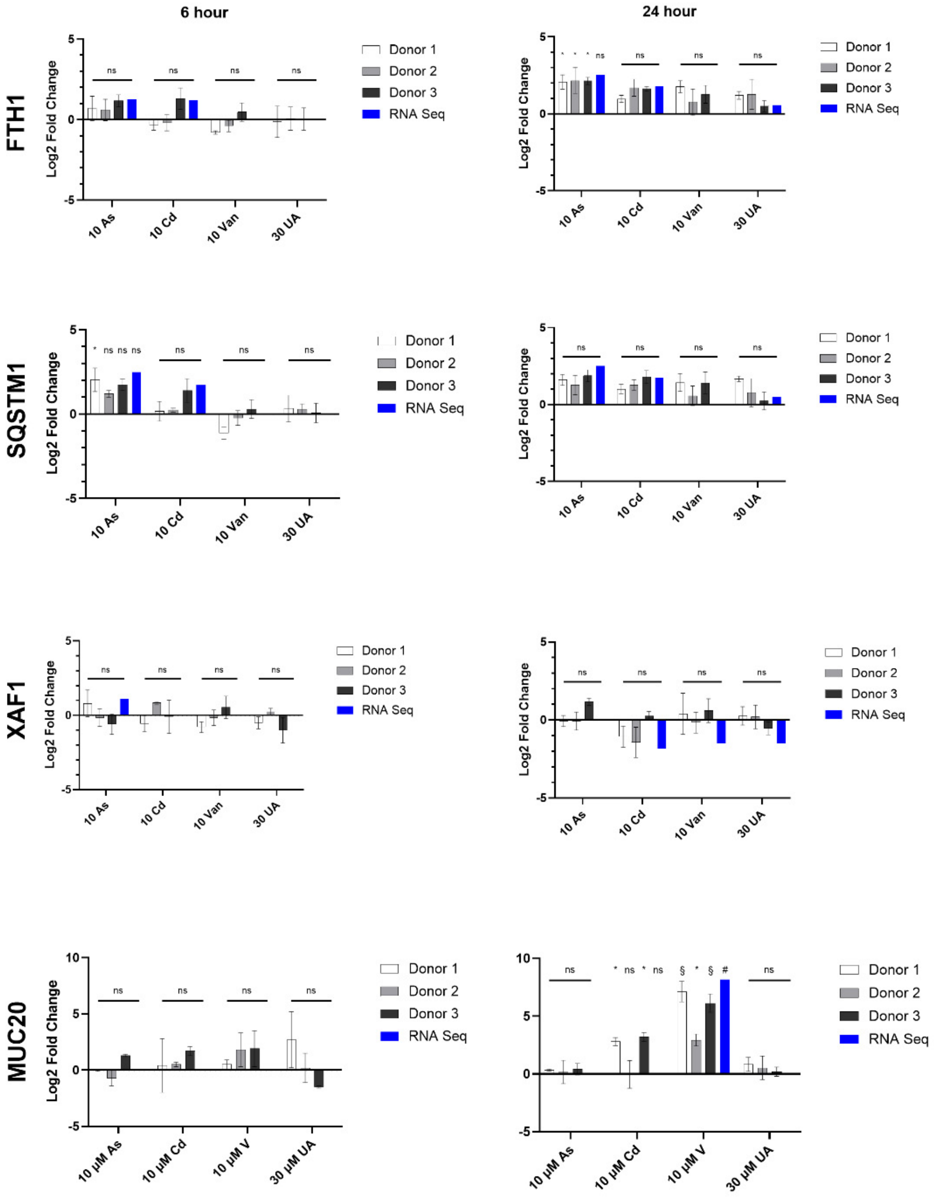

Figure S2. Comparison of log2 fold-change measured by RT-qPCR and RNA-seq for selected validation genes. RT-qPCR results are shown as mean ± SEM log2 fold-change for technical replicates (n = 3) from all donors (each normalized to the matched NT control and transformed to log2 scale). RNA-seq log2 fold-change values were calculated from Donor 1 as log2(treated/NT) using mean expression (FPKM averaged across n = 3 replicates per condition). Statistical significance for RT-qPCR was assessed by two-way ANOVA (factors: treatment and donor) followed by Tukey's multiple comparisons test; ns, not significant; * $p \leq 0.05$; † $p \leq 0.01$; # $p \leq 0.001$; § $p \leq 0.0001$.

Figure S3. PCA plot for 6 hr metal treatment.

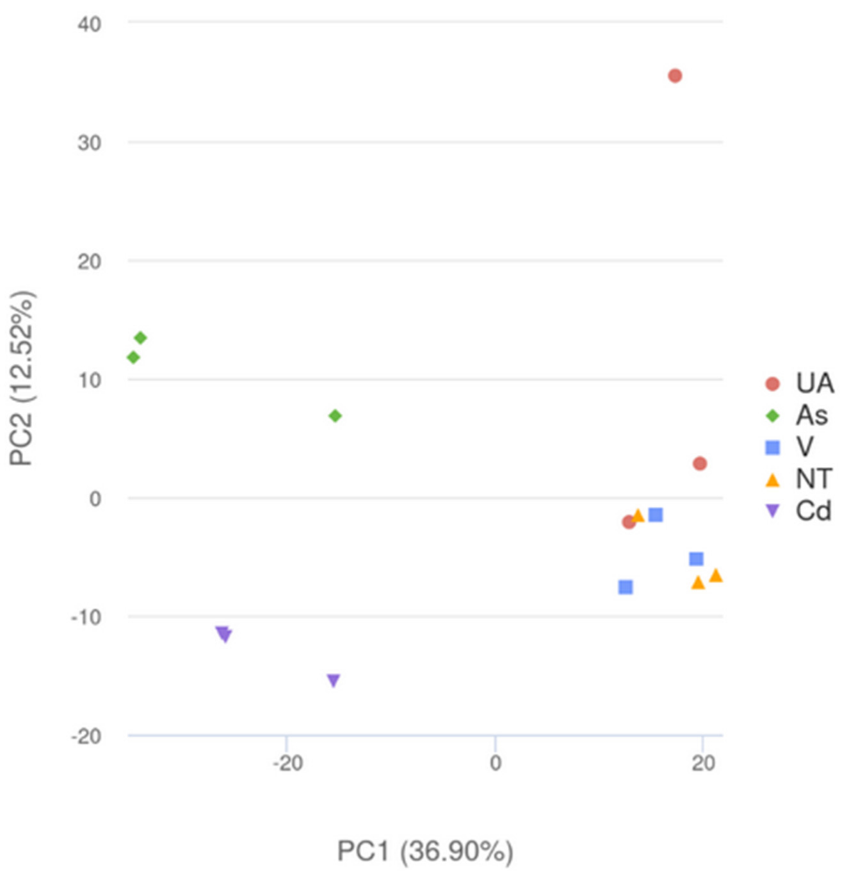

Figure S3. Principal component analysis of differentially expressed genes after 6 hr metal treatment. RPTE cells were treated with individual metals (10 μM As, 10 μM Cd, 10 μM V & 30 μM UA) for 6 hr and harvested as described under "Materials and Methods". In the principal component analysis (PCA), the first component (PC1) explains 36.90% of the variance and the second component (PC2) explains 12.52% of the variance.

Figure S4. PCA plot for 24 hr metal treatment.

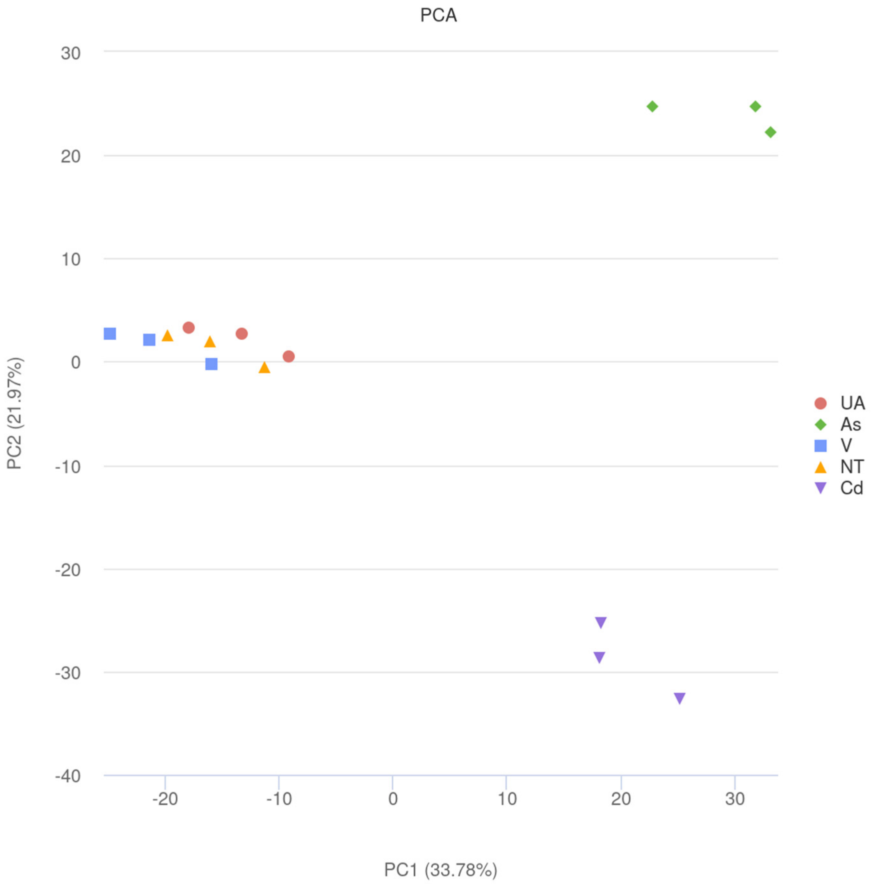

Figure S4. Principal component analysis of differentially expressed genes after 24 hr metal treatment. RPTE cells were treated with individual metals (10 µM As, 10 µM Cd, 10 µM V & 30 µM UA) for 24 hr and harvested as described under "Materials and Methods". The first component (PC1) explains 33.78% of the variance and the second component (PC2) explains 21.97% of the variance. The gene expression profiles of NT (control), UA-, and V-treated samples cluster closely on both PC1 and PC2, indicating similarity. In contrast, As- and Cd-treated samples are aligned along PC1 but diverge along PC2, suggesting shared patterns with distinct secondary variation.

Figure S5. Inductively Coupled Plasma Mass Spectrometry of RPTE cells after UA treatment.

RPTE cells were grown as described in Materials and Methods, section 2.1. Cells were grown until they were 70% confluent, then treated with UA for either 6 or 24 hours. Cells were collected using a TrypKit (Lifeline Cell Technology LL-0013) according to the manufacturer's instructions. The cells were placed into pre-weighed, acid-washed tubes and washed once in PBS, after which the supernatant was removed as close to the pellet as possible. The tubes were placed into a sealed vessel surrounded by Drierite (Drierite AA-0124) for 7 days at room temperature. The tubes were then weighed again, and the dry weight of each pellet was determined. Each

pellet was dissolved in 0.5 mL of nitric acid (Fisher NX0407-1) at room temperature overnight. Each sample was then diluted in 4.5 mL of water which had been treated with Chelex (BioRad 1422842) and filtered before use. Samples were frozen at -20°C for a minimum of 24 hours, then thawed and centrifuged at 4500 x g for 10 minutes to remove particulates. 1 mL of supernatant was added to 2 mL filtered chelexed water in a fresh acid-washed tube.

Uranium concentrations were quantified by inductively-coupled plasma mass spectrometry (ICP-MS) (Agilent 7900, USA) coupled with an SPS 4 autosampler. The ICP-MS instrument was optimized using multi-element tuning solution over a wide range of masses and calibrated using Environmental calibration standards (Part number 5183-4688, Agilent). All measurements were conducted in triplicates and in He Mode. All solutions were made with 2% nitric acid to match the standards matrix. The system was calibrated with blanks and 5 calibration standards, and samples were analyzed including quality control samples for data validation and verification. In addition, each experimental sample batch included a Continuing Calibration Verification quality control sample that was analyzed after every 10 samples to further validate the calibration and instrument stability. The settings of the Agilent 7900 ICP-MS are as follows: RF power, 1550 W; nebulizer gas,1.09 L/min; nebulizer pump, 0.1 rps; sample depth, 10 mm; S/C temp, 2C; nebulizer type, MicroMist.

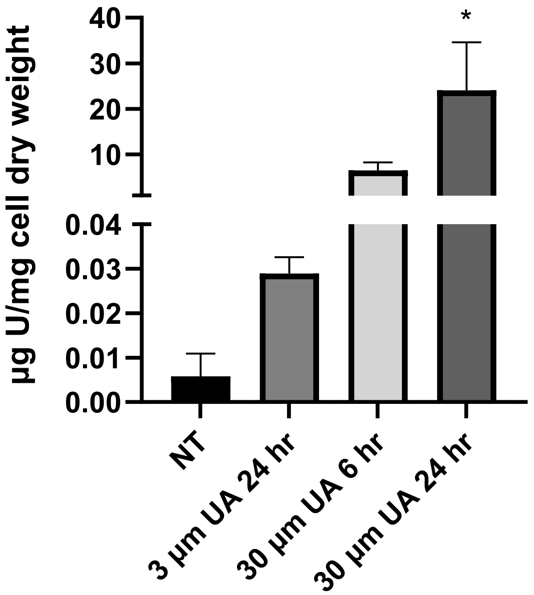

Figure S5. ICP-MS of UA in RPTE cells. Concentrations of uranium (reported as µg U/mg cell dry weight) in RPTE cells were quantified by ICP-MS. For each biological replicate (run), uranium was measured in technical triplicate, and the triplicate values were averaged to obtain a single value per run. The number of independent runs was n = 2 for 3 µM UA and n = 4 for 30 µM UA and NT. Differences between NT and 30 µM UA (24 hr) were evaluated by one-way ANOVA (* $p \leq 0.05$).
